# Automatic aggression of future sports coaches is influenced by the presence of nevi—an exploratory study

**DOI:** 10.3389/fpsyg.2026.1838708

**Published:** 2026-04-30

**Authors:** Tatiana Niță, Florin Voinea, Mihaela Zahiu, Marian Alexandru Cosma, Dan Mircea Enescu

**Affiliations:** 1 University of Medicine and Pharmacy “Carol Davila”, Institute of Doctoral Studies, Bucharest, Romania; 2 Faculty of Physical Education of Sport, Physical Education, Sport and Physiotherapy Department, Ovidius University of Constanta, Constanta, Romania; 3 National University of Physical Education and Sports, Faculty of Physical Education and Sport, Physical Education and Sports Department, Bucharest, Romania; 4 University of Craiova, Faculty of Physical Education of Sport, Craiova, Romania

**Keywords:** automatic aggression, IAT, nevi, professional development, sports coaches

## Abstract

**Introduction:**

Aggression in sport-related contexts has traditionally been examined through explicit self-report measures and situational determinants, while considerably less attention has been given to potential body-related correlates that may influence automatic aggressive tendencies. The aim of the research was to explore whether future sports coaches’ automatic (implicit) aggression relate to nevus visibility (size and number). At the same time, we checked if the presence of nevi predicts automatic aggression in future coaches.

**Materials and methods:**

Eighty-nine participants took part in the study, all male future sports coaches, aged 20–22 years. For automatic (unconscious) aggression an Implicit Associations Test (IAT) was applied, using the classic 7-block version.

**Results:**

Data analysis revealed (using analysis of variance and Tukey post-hoc test) statistically significant differences in automatic aggression between future coaches with bigger facial nevi (*p* = 0.020), having a greater number of nevi on the upper and lower limbs (*p* = 0.016) and those who did not reported nevi in these areas of the body. Also, binomial logistic regression procedures were carried out to predict future sports coaches’ likelihood of implicit aggression based on the presence of nevi across different body regions.

**Conclusion:**

The findings provide exploratory evidence regarding the relationship between automatic aggression of future sports coaches and the presence of nevi, emphasizing the potential relevance of physical appearance-related markers in shaping automatic cognitive associations related to aggression. Larger facial nevi and a greater number of nevi at limbs level were linked to stronger associations between Aggression and Others category, at the implicit, subconscious level.

## 1 Introduction

The assessment of aggression in sport psychology has traditionally relied on explicit self-report instruments. However, specialized literature emphasizes the importance of examining both conscious and unconscious processes involved in aggressive tendencies (for the dual processing model see, for example, Gawronski and Bodenhausen, 2006). Within this framework, the Implicit Association Test (IAT) has been increasingly used in sport with athletes (Teubel et al., 2011; Mascret et al., 2016; Predoiu et al., 2024a; Predoiu et al., 2024b; Gerber et al., 2019) and coaches (Predoiu et al., 2022) as a tool for measuring automatic or implicit forms of aggression that may not be captured through direct questionnaires. However, these is still a lack of studies investigating automatic aggression in sports.

Automatic processing is less visible (Todorov and Bargh, 2002), occurs in the absence of conscious control (De Houwer et al., 2009), being triggered by situational characteristics as a result of the activation from memory of different associations. Therefore, not only the explicit, direct manifestation of aggression (e.g., physical aggression, verbal aggression) is important, but also the automated (unconscious) information processing define the aggressive behavior (Richetin and Richardson, 2008).

Studies conducted in educational contexts and in sports have shown that implicit measures such as the IAT can reveal patterns of automatic associations that may differ from explicit self-reported attitudes. For instance, research investigating athletes and future teachers (Predoiu et al., 2024a) found that most participants automatically associated Aggression with Others rather than with Self (on a subconscious level). Furthermore, the IAT has been applied in sport science to explore the relationship between implicit aggression and performance-related variables. Findings indicate that athletes (in martial arts) with higher competitive achievements may show stronger automatic associations between aggression-related concepts and Others (not Self) managing, therefore, to “generate an emotional tension during trainings, closer to competitions, having embedded in their deep structures of the psychic system the information that sports performance is very difficult to be achieved” (Predoiu et al., 2024b). Also, Teubel et al. (2011) explored basketball players, emphasizing that implicit (automatic) aggression is a better predictor of sports performance than explicit/direct measures. These results suggest that implicit measures of aggression can provide valuable insights into psychological mechanisms operating beyond conscious awareness.

Within sport-related professional pathway, appearance-linked stressors (such as nevi) are especially relevant because future coaches are expected to enact high levels of social presence, authority, and interpersonal regulation. In a recent study, nevi characteristics (presence, number, and size in visible regions) were linked to heightened perceived stress in emerging future teachers (Niță et al., 2025). The present paper builds on this psychosocial dermatology evidence by proposing that nevus visibility may relate not only to self-reported stress, but also to automatic/ implicit aggression. This link is theoretically grounded in the Cognitive Neo-Association Theory, which posits that experienced negative affect activates associative networks containing anger- and aggression-related thoughts, memories, and action tendencies, thereby increasing the likelihood of aggressive responding under certain conditions (Berkowitz, 1990). If visible nevi function as chronic or situational cues of social-evaluative threat (e.g., stigma anticipation), they may contribute to negative affective states (e.g., tension, shame, intrapsychic stress), which—per Berkowitz’s model—could prime aggression-related associative content outside conscious awareness. To capture such fast, indirect processes, the paper focuses on implicit aggression assessment. Indirect reaction-time paradigms (e.g., Aggressiveness-IAT) operationalize automatically activated self-concepts of aggression and show incremental prediction of observed aggressive behavior beyond explicit self-reports (Banse et al., 2015; Kovačić et al., 2018). More broadly, the implicit-cognition literature argues that automatic processes can meaningfully contribute to aggressive behavior, particularly when controlled self-regulation is limited or when affective cues are salient (Richetin and Richardson, 2008). Methodologically, implicit paradigms such as the Affect Misattribution Procedure provide an established framework for quantifying automatically activated evaluative responses with reduced susceptibility to demand characteristics (Payne et al., 2005; Blaison et al., 2012). Taken together, these findings justify investigating whether nevus visibility (size/number) is associated with future sports coaches’ implicit aggression. We mention a gap in the literature regarding the link between the automatic aggression of sports coaches and visible cutaneous markers (e.g., nevi).

Visible markers (for example, facial nevi) can carry psychosocial meaning because they are continuously “read” in social interaction, may elicit staring, intrusive questions, or avoidance, and can become targets of self-focused attention and appearance-related concerns (Zahra et al., 2025; Masnari et al., 2022). Although medically benign, visible nevi may have psychosocial implications, particularly when they are located in prominent body areas such as the face. Research on melanocytic nevi indicates that individuals may experience social reactions including staring or teasing, which can contribute to feelings of stigmatization and may negatively affect self-esteem and quality of life (Mologousis et al., 2024). These findings highlight that skin/body characteristics can extend beyond purely medical considerations and may interact with social perception and psychological well-being. Also, contemporary research consistently shows that visibility of skin conditions is a key driver of perceived stigma and downstream outcomes (e.g., diminished self-esteem, anxiety/depressive symptoms, social withdrawal), with conceptual models emphasizing both external stigma (others’ reactions) and self-stigma (internalized devaluation) as mechanisms linking appearance to negative affect and reduced quality of life (Germain et al., 2021; Sampogna et al., 2021).

Studies examining the psychological impact of visible skin conditions suggest that individuals with noticeable dermatological features frequently report elevated levels of emotional discomfort and concerns related to body image. Such conditions may contribute to increased self-awareness during social interactions and may influence interpersonal behavior due to the anticipation of negative social reactions or judgment from others (Koonjobeeharry et al., 2025). These experiences can generate psychological strain and may influence how individuals interpret social cues and interpersonal situations. The psychosocial impact of visible skin features may also extend to professional environments that involve continuous social interaction. Professions that require leadership, communication, and public visibility—such as sports coaching—place individuals in situations where appearance-related cues can become particularly salient, emotional well-being, interpersonal confidence and social behaviors being influenced (Clarke et al., 2013). Perceived social scrutiny or appearance-related concerns can generate negative affective states such as stress, embarrassment, or anxiety. According to psychosocial models of visible difference, these emotional reactions may influence interpersonal behavior by increasing vigilance toward social cues and potentially amplifying emotional responses during social interactions (Thompson and Kent, 2001). In professional roles involving authority and interaction, such as sports coaching, these processes may indirectly influence communication style, emotional regulation, and behavioral tendencies.

Sports coaches operate in highly interactive social environments, where continuous observation and evaluation can intensify psychological pressure. Situations involving scrutiny and performance appraisal may heighten emotional strain (Harmsen et al., 2018). These challenges are often particularly salient during the early stages of the career. Research on visible physical differences suggests that noticeable appearance features may shape psychological adjustment and perceived life satisfaction (Clarke et al., 2013). Not least, such appearance-related factors can influence interpersonal dynamics of coaches (and not only), social perception, and individuals’ strategies of self-presentation within professional contexts (Rumsey and Harcourt, 2012).

### 1.1 The current study

The aim of the research is to explore whether future sports coaches’ automatic (implicit) aggression relate to nevus visibility (operationalized through size and number). At the same time, we checked if the presence of nevi predicts automatic (indirect) aggression in future sports coaches.

While aggression in sport contexts has traditionally been examined through self-report (explicit) measures and situational predictors (e.g., Conway et al., 2024; Makarowski et al., 2021; Buss and Perry, 1992), considerably less attention has been given to the potential body-related correlates of implicit aggressive tendencies. The present study fills a gap in the literature considering the relation between sports coaches’ automatic (implicit) aggression and nevi visibility.

## 2 Objectives

- Knowing the automatic aggression level in future sport coaches;
- Identifying the differences between future coaches, in terms of implicit aggression, taking into consideration the size and number of facial nevi, nevi on limbs and trunk;
- Knowing predictors of automatic aggression starting from nevus visibility.

### 2.1 Research questions

The following research questions, regarding the automatic aggression of future coaches, were put forward (given that there is limited literature on this topic and no hypotheses are formulated in such a context—McGarry et al., 2021):

1) How does the size of nevi (on the face, trunk, and limbs) influence future sports coaches’ automatic (implicit) aggression?
2) How automatic aggression of future sports coaches varies depending on the number of facial nevi, nevi at the level of limbs and trunk?
3) To what extent the presence of nevi predicts implicit (automatic) aggression in future sports coaches?

## 3 Materials and methods

### 3.1 Participants

A total of 93 participants took part in the research (in the early stages of the study), all male future sports coaches, aged 20–22 years, clinically healthy, 3rd year students at physical education and sports faculties in Romania. Participants will become in a few months (at most a year) certified sports coaches in sports disciplines with direct contact with the opponent (an inclusion criterion for the present study): soccer, martial arts, handball and basketball.

The convenience and purposive sampling techniques were used to identify and examine male future coaches in sports branches such as football, martial arts (karate, boxing, fencing, taekwondo and judo), handball and basketball (sports disciplines where there is direct contact with the opponent).

### 3.2 Measures

For automatic, latency-based measure of aggression an Implicit Associations Test (IAT) was applied, using the 20 + 40 trials subdivision (Schnabel et al., 2008), and the classic 7-block version (Greenwald et al., 1998). The IAT evaluates the strength of associations between conceptual categories by measuring reaction times during classification tasks—IAT being a computerized dual-categorization task (Sukhera et al., 2019).

In the context of aggression research in sport and the IAT, participants are required to categorize words related to aggression (in the current IAT: threat, insult, hit, beat and swear, see Predoiu et al., 2022) and non-aggression (respect, fairness, encouragement, fair play and discipline) together with self-related (I, mine, me, self, my) and other-related (theirs, them, they, their, other) stimuli. Faster reaction times when pairing certain categories indicate stronger automatic associations between those concepts (Predoiu et al., 2024a). The words specific to the following categories: Self and Others, were translated in Romanian, from previous studies (Greenwald and Farnham, 2000; Banse et al., 2015).

The IAT in the current study lasts 16 min, it was created in the free-to-use platform PsyToolkit (Stoet, 2010; Stoet, 2017), and it was used in previous research with coaches (Predoiu et al., 2022; Predoiu et al., 2025), with athletes from team sports and individual sports (Predoiu et al., 2024a; Predoiu et al., 2024b). In each of the 7 blocks of the IAT “the task of the participants was to sort the stimuli into categories by pressing the E or I key on the keyboard” (Predoiu et al., 2022), as fast and accurately as possible. For example, when categories Non-Aggression and Self (upper right) and Aggression and Others (upper left) were presented on the computer screen, in the center of the monitor the words/attributes representative for these categories (Aggression, Self, Non-Aggression or Others) were displayed, while participants had to quickly sort the attributes into the correct category.

The improved D-scoring algorithm was used to calculate the score on the Implicit Association Test, labeled as D_4_ (see Greenwald et al., 2003). With respect to D_4_, “in the case of an error, it was replaced with: mean(C) + 600 ms, where mean(C) is the block mean of correct-response latencies” (Predoiu et al., 2024b). A positive D-score translates into a stronger association between Aggression and Self, while a negative D-score emphasizes a stronger link between Aggression and Others.

To investigate the socio-demographic data in the case of future sports coaches, and the presence of facial nevi, nevi on the trunk (anterior, posterior), and on the upper and lower limbs, both close- and open-ended questions were used (nevi number and size were asked). Item examples: *Mention the number of facial nevi* (open question); *Mention the size of facial nevi (select the answer taking into consideration the largest nevus)*—ways of answering: “I do not have facial nevi,” “below 5 mm,” “between 5 and 10 mm,” “between 10 and 20 mm,” “over 20 mm.” Therefore, regarding the size of nevi (in various regions of the body) the largest dimension of the nevi was reported.

### 3.3 Procedure

In the early stages of the study 93 eligible future coaches (in terms of gender and sports discipline) completed the IAT, but 89 remained for future statistical analysis. Four participants were excluded from the study having D scores (in absolute value) > 0.65 (see Klein, 2020), in order for the confidence intervals (in the case of D scores) to span below the 0.65 cutoff, meaning a slight or moderate bias (not a strong bias)” (Predoiu et al., 2022). No participant was removed from the statistical analysis for exceeding the error rate of 35% in one of the blocks in the Implicit Association Test (see Banse et al., 2015).

The investigation was carried out over the course of two academic years (between October 2024 and January 2026). The research was conducted in Romania. Participants completed the IAT in their free time on a computer (the test cannot be performed on a phone), with the help of the platform called PsyToolkit (see Stoet, 2010; Stoet, 2017). The questionnaire (including age, gender and the items concerning the size and number of nevi across multiple body regions) was applied via *Google forms* (Google LLC, Mountain View, CA, United States). Clarifications regarding nevi were provided by the experimenter when distributing the questionnaire to the participants. Also, information about nevi was provided at the beginning of the questionnaire (the same as IAT, the questionnaire was completed by the future coaches in their free time). The current study is based on ex post facto design (Thomas and Nelson, 2001).

### 3.4 Statistical analysis

Jamovi (The Jamovi Project, 2024, Version 2.6) and IBM SPSS Statistics Version 27.0 (Armonk, NY, IBM Corp) were used for the statistical analysis. In the case of analysis of variance, Tukey post-hoc test was performed due to Levene’s test outcome (*p* > 0.05, the assumption of homogeneity of variances being met)—AlRyalat and Momani (2019). Skewness and Kurtosis coefficients were verified to ensure that the normality condition (in the case of the D-scores) was met. Variables were normally distributed, Skewness and Kurtosis coefficients ranging within ±2 (George and Mallery, 2010). Cohen’s *f* (the effect size for ANOVA) was interpreted as follows: 0.10 = small effect size, 0.25 = medium, while 0.40 = large effect size (Hahs-Vaughn and Lomax, 2020). Also, binomial logistic regression was used, with the following effect size index (Nagelkerke *R*^2^) interpretation: 0.02 small effect size, 0.35 large effect size, 0.15 medium effect size (Cohen, 1992). Nagelkerke *R*^2^ is preferred in the case of logistic regression, being an adjusted version of the Cox and Snell *R*^2^, ranging from 0 to 1 (Bewick et al., 2005).

## 4 Results

Data analysis (box-plot and stem-and-leaf) revealed no outliers in the case of D-scores (IAT). Future sports coaches in the current research obtained negative D-scores, meaning that they automatically (subconsciously) associated Aggression with Others (not with Self). This *pattern* was observed in prior research using the IAT (Predoiu et al., 2022; Predoiu et al., 2024a, 2024b) when martial arts coaches and athletes were explored, maybe due “to the words designated as representative for aggression in sports […] (threat, beat, hit, swear and insult)”, which are, generally, closer to a more hostile (and not instrumental) form of aggression (Predoiu et al., 2022).

First, the way future sports coaches’ automatic (indirect) aggression is influenced by the size and number of nevi was examined. The normality of score distributions was evaluated using the Skewness and Kurtosis coefficients (in absolute value), which were less than 2, the assumption of normality being met for the dependent variable (D-scores), in each investigated group (taking into consideration the size and number of nevi in multiple body regions: face, trunk and limbs). Also, the assumption of homogeneity of variances (Levene’s test) across groups was met – specifically, non-significant values were obtained for D-scores (IAT): *F* = 1.03, *p* = 0.360 (size of facial nevi), *F* = 0.528, *p* = 0.664 (number of facial nevi), *F* = 1,05, *p* = 0.376 (number of nevi on upper and lower limbs), *F* = 1.02, *p* = 0.352 (size of nevi − upper and lower limbs), *F* = 1.31, *p* = 0.277 (number of nevi on the anterior and posterior trunk), respectively *F* = 1.05, *p* = 0.353 (size of nevi − anterior and posterior trunk).

Variations in automatic aggression among future sports coaches were examined according to the size and number of facial nevi, nevi located on the upper and lower limbs, as well as on the trunk (anterior and posterior). Separate one-way ANOVAs were conducted, in order to answer the first two research questions. The analysis (presented in Table 1) revealed a statistically significant effect for automatic aggression, indicating that levels of implicit aggression in future coaches varied significantly depending on the size of facial nevi—*F*(2,86) = 4.27, *p* = 0.017, and on the number of nevi on upper and lower limbs—*F*(3,85) = 4.19, *p* = 0.008. In contrast, no significant differences were observed for D-scores (IAT), when analyses were conducted for the number of facial nevi (*p* = 0.090), the size of nevi on upper and lower limbs (*p* = 0.883), and for nevi located on the trunk (*p* = 0.471 − size of nevi, respectively *p* = 0.510 − number of nevi).

**Table 1:** One-way ANOVA (Fisher’s)—automatic aggression and the presence of nevi in future sports’ coaches.

Variable	*F*	df1	df2	*p*
D-scores—automatic aggression of future sports coaches	Size of facial nevi			
	4.27	2	86	0.017
	Number of facial nevi			
	2.23	3	85	0.090
	Upper and lower limbs—size of nevi			
	0.124	2	86	0.883
	Upper and lower limbs—number of nevi			
	4.19	3	85	0.008
	Trunk (anterior and posterior)—size of nevi			
	0.760	2	86	0.471
	Trunk (anterior and posterior)—number of nevi			
	0.778	3	85	0.510

Table 2 (post-hoc analysis) reports only the results for the significant differences found between groups.

**Table 2:** Tukey post-hoc test—future sports coaches’ indirect/latency-based measure of aggression depending on the size and number of the nevi.

Automatic aggression of sports coaches—size of facial nevi				
IAT and size of facial nevi		I do not have nevi	Below 5 mm	Between 5 and 10 mm
I do not have nevi	Mean difference	–	0.135	0.180
	*p*-value	–	0.040	0.020
Below 5 mm	Mean difference		–	0.045
	*p*-value		–	0.677

Automatic aggression of sports coaches—number of nevi on upper and lower limbs					
IAT and number of nevi		I do not have nevi	1–2	3–5	Minimum 6
I do not have nevi	Mean difference	–	0.164	0.170	0.078
	*p*-value	–	0.015	0.016	0.764
1–2	Mean difference		–	0.006	−0.086
	*p*-value		–	0.999	0.686
3–5	Mean difference			–	−0.092
	*p*-value			–	0.651

In Table 2, post-hoc Tukey analyses further clarified the significant ANOVA effect observed for implicit (automatic) aggression. A statistically significant difference emerged between future sports coaches with facial nevi below 5 mm (mean difference = 0.135, *p* = 0.040), between 5 and 10 mm (mean difference = 0.180, *p* = 0.020) and those who did not reported facial nevi. Cohen’ *f* (effect size) is 0.27 and 0.30 respectively, emphasizing a moderate, respectively and a moderate to strong difference between groups. Also, a significant difference is observed between future coaches having a greater number of nevi on the upper and lower limbs (1–2 nevi, mean difference = 0.164, *p* = 0.015, respectively 3–5 nevi, mean difference = 0.170, *p* = 0.016), and those who did not reported nevi in these areas of the body. The effect size index (*f* = 0.35) revealed a moderate to strong difference between groups, in terms of automatic aggression (for both comparisons). No meaningful between-group differences were observed for automatic aggression depending on nevi (size and number) on future coaches’ trunk (anterior and posterior), all Tukey comparisons being non-significant (*p* > 0.05).

Results in Table 3 (descriptive statistics) indicate a higher level of automatic aggression (Aggression associated with Others, not with Self) in future sports coaches with larger facial nevi, and having a greater number of nevi on the upper and lower limbs (in the case of future coaches having minimum 6 nevi on upper and lower limbs, the reduced sample size greatly limits the findings). Mean scores increased progressively with nevus size (facial nevi), and with nevus number (on upper and lower limbs) with the lowest values observed in future sports coaches who did not report facial nevi (*M* = 0.249, *SD* = 0.19) or nevi on upper and lower limbs (*M* = 0.253, *SD* = 0.195), and the highest scores among future sports coaches presenting facial nevi between 5–10 mm (*M* = 0.43, *SD* = 0.175) or 3–5 nevi on upper and lower limbs (*M* = 0.423, *SD* = 0.184, see Table 3).

**Table 3:** Descriptive statistics—implicit (automatic) aggression of future sports coaches (*N* = 89).

Facial nevi				
Size	*N*	Mean—automatic aggression	SD	SE
I do not have nevi	18	−0.249	0.19	0.044
Below 5 mm	52	−0.385	0.21	0.029
Between 5–10 mm	19	−0.43	0.175	0.04
Number	*N*	Mean—automatic aggression	SD	SE
I do not have nevi	18	−0.264	0.186	0.045
1–2	33	−0.362	0.206	0.035
3–5	18	−0.419	0.187	0.044
Minimum 6	20	−0.413	0.222	0.048

Upper and lower limbs				
Number	*N*	Mean—automatic aggression	SD	SE
I do not have nevi	24	−0.253	0.195	0.039
1–2	31	−0.417	0.191	0.034
3–5	26	−0.423	0.184	0.036
Minimum 6	8	−0.331	0.257	0.091
Size	*N*	Mean—automatic aggression	SD	SE
I do not have nevi	24	−0.382	0.201	0.036
Below 5 mm	46	−0.357	0.202	0.029
Between 5 and 10 mm	19	−0.365	0.252	0.072

Trunk (anterior and posterior)				
Number	*N*	Mean—automatic aggression	SD	SE
I do not have nevi	14	−0.306	0.214	0.057
1–2	35	−0.384	0.191	0.032
3–5	18	−0.407	0.192	0.045
Minimum 6	22	−0.346	0.239	0.05
Size	*N*	Mean—automatic aggression	SD	SE
I do not have nevi	14	−0.306	0.214	0.057
Below 5 mm	64	−0.381	0.197	0.024
Between 5 and 10 mm	11	−0.361	0.256	0.077

Although the results of the Tukey post-hoc test did not reveal any significant differences between the groups under investigation (*p* > 0.05) based on the number of facial nevi and nevi on the trunk, a higher level of implicit aggression can be observed in future coaches (Aggression associated with Others, at the automatic, subconscious level) as the number of facial nevi increases, as well as the number of nevi on the trunk (see Table 3).

In a second phase, the extent to which nevi predict implicit (automatic) aggression in future sports coaches was explored. Two separate binomial logistic regressions were performed (Tables 4–6), with size of facial nevi and number of nevi on upper and lower limbs playing the role of the independent variables (predictors). Regarding the size of facial nevi: 1 = I do not have nevi, 2 = nevi below 5 mm, respectively 3 = nevi between 5 and 10 mm (*N* = 89), while for nevi on upper and lower limbs: 1 = I do not have nevi, 2 = 1–2 nevi, respectively 3 = 3–5 nevi (*N* = 81). The future sports coaches reporting minimum 6 nevi at limbs level were removed from the current statistical analysis due to their reduced number (*N* = 8) while, also, *p* = 0.764 when their automatic aggression level was compared to that of future sports coaches who did not report nevi on upper and lower limbs.

**Table 4:** Binomial logistic regressions analysis—automatic aggression of future sports coaches as criterion.

Size of facial nevi predicting implicit (automatic) aggression of future sports coaches	
Omnibus likelihood ratio test: Chi-square and *p* values	*χ*^2^ = 4.65 and *p* = 0.031
Nagelkerke *R*^2^ (effect size)	0.068
Overall percentage (Predicted − Percentage correct)	65.2%—Accuracy
D-scores between 0.367 and 0.65 (predicted)	90%—Specificity
D-scores between 0.004 and 0.366 (predicted)	33.3%—Sensitivity

Number of nevi on upper and lower limbs predicting automatic aggression of future coaches	
Omnibus likelihood ratio test: Chi-square and *p* values	*χ*^2^ = 6.55 and *p* = 0.010
Nagelkerke *R*^2^ (effect size)	0.104
Overall percentage (Predicted − Percentage correct)	69.1%—Accuracy
D-scores between 0.367 and 0.65 (predicted)	84.8%—Specificity
D-scores between 0.004 and 0.366 (predicted)	48.6%—Sensitivity

**Table 5:** Variables in the equation—size of facial nevi (predictor) and sports coaches’ automatic aggression.

Variables						95% confidence interval	
Predictor	Estimate	SE	*Z*	*p*	Odds ratio	Lower	Upper
Intercept	1.223	0.733	1.67	0.095	3.398	0.808	14.293
Size of facial nevi	−0.738	0.354	−2.09	0.037	0.478	0.239	0.956

**Table 6:** Variables in the equation—number of nevi on upper and lower limbs (predictor) and future sports coaches’ automatic aggression.

Variables						95% confidence interval	
Predictor	Estimate	SE	*Z*	*p*	Odds ratio	Lower	Upper
Intercept	1.235	0.645	1.91	0.056	3.439	0.971	12.181
Number of nevi (limbs level)	−0.756	0.306	−2.47	0.013	0.469	0.258	0.855

The mean was calculated for the automatic aggression of future sports coaches (at group level, *N* = 89)—for the current study *M* = −0.367. With respect to Blanton et al. (2015) research, an arbitrary character was emphasized regarding the cutoff criteria for D-scores, therefore we will discuss about a stronger or a weaker association between Aggression and Others (the same as Predoiu et al., 2024b), in the present study the categorizations of D-scores (in order to perform the binomial logistic regressions analysis) being: 1 = D-scores (in absolute values) between 0.367 and 0.65 (meaning a stronger association between Aggression and Others) and 0 = D-scores (in absolute values) between 0.004 and 0.366 (indicating a weaker association between Aggression and Others, on a subconscious level).

The models are significant (*p* = 0.031, respectively *p* = 0.010, see Omnibus likelihood ratio test, Table 4). The models correctly classified 65.2% (size of facial nevi and implicit aggression of future coaches), respectively 69.1% (number of nevi on upper and lower limbs and automatic aggression) of cases. The effect size index (Nagelkerke *R*^2^) highlights a moderate to weak relation (*R*^2^ = 0.068, respectively, *R*^2^ = 0.10) between the presence of nevi and the automatic aggression of future sports coaches.

In the case of future coaches, the size of facial nevi and the number of nevi at the level of upper and lower limbs represent important predictors of automatic (implicit) aggression, a valuable resource for sports psychologists and for young coaches in their process of personal and professional development. Bigger facial nevi (between 5 and 10 mm) and more nevi at limbs level (generally, between 3 and 5) are associated with an increased likelihood of automatic aggression in future coaches, but Aggression associated with Others (at implicit/subconscious level), not with Self.

## 5 Discussion

The present study sought to extend the current understanding of aggression in sport-related contexts by examining whether visible skin characteristics among future sports coaches (the presence of nevi) are associated with automatic (implicit) aggression. While aggression in sport psychology has predominantly been investigated through explicit self-report measures, considerably less attention has been devoted to potential appearance-linked correlates that may influence implicit aggression. By integrating the presence of nevi (size and number) with a latency-based measure of aggression (IAT), the study aimed to explore whether physical markers of future sports specialists may relate to automatic cognitive associations involving aggression.

Data analysis revealed a statistically significant effect for implicit aggression of future sports coaches, indicating that levels of automatic aggression (in future coaches) varied significantly depending on the size of facial nevi, and on the number of nevi on upper and lower limbs. In contrast, no significant differences were found in terms of implicit aggression of future specialists when number of facial nevi, the size of nevi on upper and lower limbs, and nevi located on the trunk (anterior and posterior) were examined. Post-hoc analyses further clarified the significant effects observed for automatic aggression—future coaches with facial nevi below 5 mm and between 5–10 mm and, also, future coaches having a greater number of nevi on the upper and lower limbs (3–5 nevi) associated at a higher level Aggression with Other (compared to future specialists who did not reported facial nevi or nevi at limbs level). Therefore, words specific to aggression (in the current IAT): swear, threat, insult, hit, beat were associated on a subconscious, automatic level with Other category (not with Self). Taking into consideration the psychosocial models of visible difference, the emotional reactions and perceived stress may influence an individual’s interpersonal behavior, potentially amplifying his/her reactions during various interactions (Thompson and Kent, 2001). Future sports coaches having larger facial nevi and more nevi on upper and lower limbs may perceive others as more aggressive and dangerous (on a subconscious level), fearing criticism based on their physical appearance (an aspect deeply rooted in the psyche). Therefore, nevi located on exposed body areas may represent subtle physical markers associated with psychological processes involved in social perception, stress responses, and implicit cognitive associations related to aggression. However, any potential associations between nevi visibility or distribution and future sports coaches’ behaviors in a given situation should be interpreted as context-dependent rather than inherently detrimental, a person’s available resources being an essential aspect, allowing us to understand why some specialists experience significant strain while others maintain psychological resilience under similar stress conditions (Bakker and Demerouti, 2017). Individuals’ reactions are shaped by multiple personal, interpersonal, and organizational determinants, suggesting that appearance-related concerns—such as self-consciousness or increased monitoring of visible characteristics—may become particularly relevant when they interact with already demanding environments (Kyriacou, 2011).

The extent to which nevi predict automatic aggression in future sports coaches was, also, explored in the present study. Larger facial nevi (between 5 and 10 mm) and more nevi at limbs level (generally, between 3 and 5) are associated with an increased likelihood of automatic aggression in future coaches, but Aggression associated with Others (at implicit/subconscious level), not with Self. These data represent valuable resources for sports psychologists and for young coaches in their process of personal and professional development.

The findings of the current study provide preliminary evidence suggesting that nevus characteristics located in visible anatomical areas (face and on upper limbs, mainly) may be associated with variations in automatic aggression patterns. In particular, the results indicate that a greater number of nevi at the level of the upper and lower limbs, and bigger facial nevi were associated with stronger automatic associations between Aggression and Others. Although the magnitude of these effects remained moderate to weak, the direction of the relationships suggests that visible skin features may represent contextual cues capable of interacting with psychological processes involved in implicit social cognition. This is very important connection to be established, between future sports coaches automatic (indirect) aggression and the presence of nevi, taking into consideration the Cognitive Neo-Association Theory which argues that experienced negative affect (and it was found that future teachers having bigger facial nevi registered higher levels of intrapsychic stress, see Niță et al., 2025) activates associative networks related to aggression, increasing people’s aggressive responses under certain circumstances (Berkowitz, 1990). It is worth mentioning, also, that a higher level of implicit aggression was observed in future coaches as the number of facial nevi increases, as well as the number of nevi on the trunk (however, the results were statistically insignificant).

In a prior study on future teachers, larger facial nevi were associated with higher levels of intrapsychic stress (Niță et al., 2025). This pattern aligns with adjustment models of visible difference, which suggest that psychosocial consequences arise primarily through social-evaluative mechanisms and heightened self-consciousness, rather than through the medical severity of the condition itself (Thompson and Kent, 2001). In the present study, we also observed that larger facial nevi were associated with stronger implicit links between Aggression and Others (not with Self). Therefore, the size of nevi, particularly at the level of the face, appears to be associated with certain psychological phenomena, such as higher levels of stress and higher automatic aggression. Evidence from large European investigations further indicates that skin concerns often coexist with psychological burden, reinforcing the importance of considering psychological outcomes in dermatological contexts (Dalgard et al., 2015).

From a practical perspective, these findings may justify the development of targeted support strategies for future specialists in sports science field (and not only) at different career stages –aimed at addressing psychosocial vulnerabilities associated with visible physical characteristics. Career stage appears to represent an important moderating factor. Early-career sports coaches and teachers frequently report elevated levels of stress related to workload and professional role expectations, and these stressors may influence a person’s practices (Fantilli and McDougall, 2009; Harmsen et al., 2018). In such circumstances, any additional psychosocial pressures—including those linked to visible appearance characteristics—may be more salient during the early phases of professional identity development.

Recent findings indicate, also, that novice sports coaches (from various sports disciplines) “who spend more time for their professional growth register higher levels of implicit aggression (Aggression associated with Others)” (Predoiu et al., 2025). However, the study of automatic processing of sports coaches’ is far from complete. Overall, the use of the IAT in sport psychology research offers an important methodological complement to explicit assessments of aggression. By capturing automatic cognitive associations, this instrument allows researchers to better understand the subtle psychological processes that may influence behavior in competitive and educational sport settings.

## 6 Limitations and directions for future research

Several limitations of the present study should be acknowledged. First, the sample was relatively homogeneous in terms of age, as it consisted primarily of university students preparing for careers in sport coaching. This relatively narrow age range may limit the generalizability of the findings, as psychological processes related to automatic aggression may vary across different stages of adulthood. Future research should therefore consider including participants from broader age groups, such as experienced coaches or professionals working in sport organizations, in order to examine whether the observed associations between visible skin characteristics and automatic (implicit) aggression remain consistent across different developmental and professional stages.

Another limitation concerns the gender distribution of the sample. Only male future sports coaches participated in the present study, which restricts the ability to generalize the findings to female populations. Psychological processes related to aggression, social perception, and responses to appearance-related cues may differ between men and women due to both socialization patterns and gender-related differences in body image, self-presentation, and sensitivity to social evaluation. Consequently, the present findings should be interpreted with caution when considering broader populations of educators or sport professionals. Interestingly, it seems that no significant differences were found “between males and females in implicit aggression” (Hu et al., 2025)—however, students were investigated in the mentioned study (not sports coaches). Moreover, previously research revealed “no significant differences between male and female coaches in terms of automatic aggression” (Predoiu et al., 2025).

Also, the reduced sample size of future sports coaches having minimum 6 nevi on upper and lower limbs limits the current findings. Further research should take into consideration larger samples of participants with more nevi in different visible body regions, including at the neck level, while nevi on upper and lower limbs, as well as nevi on anterior trunk and posterior trunk should be investigated separately. Future coaches should be examined, also, separately, according to the sports discipline practiced, sports branches with or without a direct contact with the opponent.

In the current study sports coaches reported the presence of nevi (their size and number) in various areas of the body using a self-assessment tool. Further studies should be, also, conducted with specialists providing a direct assessment of the nevi. However, it is worth arguing that self-report tools were used in many studies which investigate medical conditions (for example, injury severity—e.g., Williams et al., 2020; Patenteu et al., 2023; Roșu, 2022). Furthermore, self-assessment tools are critical tools for identifying patients with various symptoms (Rădoi et al., 2019).

With respect to the Implicit Association Test (IAT) used, the representative words for Aggression in sport and Non-aggression in sport, respectively, are influenced by specific settings (e.g., country), therefore other results could be obtained if other IATs (measuring aggression and including different attributes/words) would be applied in various countries. Also, other methods of measuring automatic aggression could be used in further studies, for example Picture Story Exercise and Conditional Reasoning Test. Also, testing different mediating mechanisms (designed to identify intermediate pathways), or using multinomial logistic regression (with three or more categories), based on one or even more independent predictor variables requires further studies.

Not least, future research should adopt longitudinal cross-lagged panel designs to better examine the relationships among the variables, to determine how the presence of nevi temporally precede the automatic (implicit) aggression of sports coaches and, also, if the emotional strain and perceived stress (which may be linked to higher levels of automatic aggression) influences a person’s physical appearance, impacting skin health.

## 7 Conclusion

The present study provides exploratory evidence regarding the relationship between automatic (implicit) aggression of future sports coaches and the presence of nevi. By examining nevus visibility through both size and number across different body regions, the research highlights the potential relevance of physical appearance-related markers in shaping automatic cognitive associations related to aggression. The results indicate that a higher number of nevi located on the upper and lower limbs was associated with stronger implicit associations between Aggression and Others (not Self). In a similar manner, larger facial nevi were also linked to stronger associations between Aggression (the words: insult, threat, swear, beat and hit) and Others category, at the implicit, subconscious level. Although these associations were moderate to weak and should be interpreted cautiously due to the exploratory nature of the study, they suggest that visible skin characteristics in future sports coaches may be related to subtle cognitive processes related to aggression. From a broader perspective, these findings contribute to expanding the understanding of aggression in sport-related educational environments by highlighting the importance of examining the automatic psychological processes. Considering that sports coaches operate in highly interactive environments that require effective emotional regulation and interpersonal communication, understanding the factors associated with automatic (implicit) aggression may be relevant for both coach education and psychological assessment.

## Data Availability

The raw data supporting the conclusions of this article will be made available by the authors, without undue reservation.
